# From Stress to Psychopathology: Relationship with Self-Reassurance and Self-Criticism in Czech University Students

**DOI:** 10.1007/s11469-021-00516-z

**Published:** 2021-03-10

**Authors:** Yasuhiro Kotera, Jaroslava Dosedlova, Denise Andrzejewski, Greta Kaluzeviciute, Makoto Sakai

**Affiliations:** 1 Human Sciences Research Centre, University of Derby, Derby, UK; 2 Department of Psychology, Masaryk University, Brno, Czech Republic; 3 Department of Psychology, Middlesex University, Dubai, United Arab Emirates; 4 School of Psychology, Chukyo University, Nagoya, Japan

**Keywords:** Stress, Self-inadequacy, Self-hate, Self-reassurance, Psychopathology, Czech university students

## Abstract

Psychological stress has become a major concern, potentially leading to diverse health problems including psychopathology such as depression and anxiety. Transactional Model of Stress and Coping is an established model, conceptualizing stressful experiences via person–environment relationship. This cross-sectional study aimed to explore the pathway from stress to depression/anxiety, with a focus on self-criticism (inadequate-self and hated-self) and self-reassurance (reassured-self) in Czech students who suffered from high prevalence of mental health problems. Convenience sample of 119 undergraduates completed the Depression Anxiety and Stress Scale-21 and the Forms of the Self-Criticizing/Attacking & Self-Reassuring Scale. Correlation and path analyses were conducted. The Strengthening the Reporting of Observational Studies in Epidemiology guidelines were used to aid an accurate and complete report of the study. Depression, anxiety, and stress were positively associated with inadequate-self and hated-self while negatively associated with reassured-self. Both inadequate-self and hated-self partially mediated the stress–depression and stress–anxiety relationships, whereas reassured-self only partially mediated the stress–depression relationship. Inadequate-self had greater impact on the stress–depression/anxiety pathways than hated-self and reassured-self. Findings indicate that clinical treatment may benefit from targeting the feelings of inadequacy to prevent stress progressing to psychopathology. This is particularly relevant as stress levels are rising globally. Our findings offer developments to the Transactional Model, and help practitioners and educators identify solutions to protect mental health of Czech university students.

While psychological stress (hereafter “stress”) is a normal part of life, it has become a major global health concern and has been identified as a notable cause for diverse health problems such as diabetes (Nyberg et al. 2014), cardiovascular disease (Iob and Steptoe 2019), fatigue (American Institute of Stress 2014), and depression (Stroud et al. 2008), and can potentially lead to self-harm (O’Connor et al. 2012) and suicidal thoughts (Liu and Miller 2014; Shahar et al. 2020). Statistics worldwide show that stress has become a pervasive pattern among college students and adults (American Psychological Association 2019; Statista 2020; YouGov 2018). In addition, the recent COVID-19 pandemic poses an imminent threat accompanied by unprecedented public health measures, which became a major cause for elevated levels of stress, anxiety (American Psychological Association 2020; Belen 2020; Montano and Acebes 2020), and depressive symptoms (Gotlib et al. 2020; Sønderskov et al. 2020). The link between stress and psychopathology has been well established and, for example, one form of psychopathology, major depressive disorder, is expected to be a leading reason for disability by 2030 (Harvard Health Publishing 2019; Yang et al. 2015) although the underlying mechanisms remain to be elucidated.

Subsequently, with stress playing such a major role in physical and mental health, several theories and models to appraise stress have been developed. One of the most prominent models is the Transactional Model of Stress and Coping (Lazarus and Folkman 1984). This model takes a person–environment approach to stress and has been actively utilized in the modern mental health studies (Kilian et al. 2021; Pflügner et al. 2020) including COVID-19 research (Trougakos et al. 2020). In their seminal stage model, Lazarus and Folkman (1984) suggest that individuals first appraise a given stressor as harmful, threatening, or challenging, and then evaluate whether sufficient resources are available to cope with the situation, which in turn determines the level of distress they are experiencing (Cohen et al. 2016; Krohne 2001). In this sequential view of the stress process, maladaptive appraisals and coping mechanisms are crucial factors in explaining the link between psychopathology and stress (Kobayashi et al. 2020). Therefore, understanding mechanisms and behaviors that affect appraisal and coping is pivotal in the prevention of mental health issues and the promotion of global mental health (Jenkins 2019; Kirmayer and Pedersen 2014).

One construct that has been strongly linked to psychopathology is self-criticism (Blatt et al. 1982; McIntyre et al. 2018). Self-criticism has been conceptualized as a depressogenic and suicidogenic personality disposition, marked by feelings of inferiority and inadequacy (Gilbert et al. 2004; Shahar et al. 2020; Werner et al. 2019). Gilbert et al. (2004) argue that there is an interaction between criticism and reassurance within the self, and suggest that self-criticism is two-dimensional: (1) hated-self that is marked by disgust and contempt toward the self, and (2) inadequate-self that is characterized by dominant coercion of a subordinate to act from oneself.

In contrast, self-reassurance (i.e., ability to focus on one’s positives and be compassionate toward self when things go wrong) functions as a buffer against self-criticism and therefore appears to be a protective factor against the development of psychopathology (Gilbert et al. 2004; Werner et al. 2019). In the context of the Transactional Model of Stress and Coping (Lazarus and Folkman 1984), it would therefore be interesting to explore the role that self-criticism plays in the progression from stress to psychopathology. Studies investigating the association between self-criticism and psychopathology have predominantly featured clinical samples (McIntyre et al. 2018; Werner et al. 2019), whereas the mediating role of self-criticism between stress and psychopathology has so far been explored only in the context of terrorism-related perceived stress (Lassri et al. 2013). To date, few studies on this topic have been conducted with non-clinical samples.

Poor mental health of the general public has become a cause of concern in the Czech Republic (Formánek et al. 2019). For example, the highest alcohol consumption globally has been found in Central and Eastern Europe (Gmel et al. 2013). Findings from the recent CZEch Mental health Study (CZEMS) indicate that alcohol dependency in the Czech Republic (6.6% vs. 3.4%) was almost twice as high as in Central Europe (Formánek et al. 2019). Strong associations between alcohol dependency and depression/anxiety have been found in several studies (Kuria et al. 2012; Smith and Randall 2012). The highest prevalence rates of alcohol dependence (16.64%), mood (7.96%), and anxiety disorders (5.42%) were found in the age group spanning between 18 and 29 years, overlapping with the age of many undergraduate university students (Formánek et al. 2019). Indeed, Czech university students’ poor mental health was identified in an empirical study, including high levels of depression and anxiety (Dosedlová et al. 2016). However, the mechanism of their mental health in relation to self-criticism and self-reassurance has not been evaluated in depth to date, suggesting a need for close evaluation.

Accordingly, the current study aims to evaluate how much one’s level of self-criticism (i.e., inadequate-self and hated-self) and self-reassurance impacts the relationship between stress and psychopathology, depression and anxiety in particular, in Czech university students. Two research questions were established:

1. How much does self-criticism impact the relationship between stress and depression/anxiety?
2. How much does self-reassurance impact the relationship between stress and depression/anxiety?

## Methods

### Participants

All participants were aged 18 years or older and were undergraduate psychology students studying at a Czech university in Brno. Students who were taking a study break were excluded from the study. Participants were recruited using convenient sampling via announcements distributed by program tutors in February 2020 (no course credits or other forms of compensation were awarded for participation).

A total of 130 students agreed to participate, of which 119 (93 females, 20 males, and 6 did not answer) completed two self-report measures, satisfying the required sample size of 115 based on statistical power calculations (Faul et al. 2009). The age range was 19 to 44 years (*M* = 21.87, SD = 3.32). The majority of students were Czech (*n* = 98), and the rest were Slovakian students (*n* = 21). The demographic information of our sample was similar to the general population of psychology students in the Czech Republic (Czech Ministry of Education 2019). The withdrawn 11 students did not give any reason nor complaint. Ethical approval was granted by the University [NAME]’s review board. Should students felt distress during the study, contact information of the student wellbeing center was provided. Informed consent was obtained from all participants in the study. This study followed the Strengthening the Reporting of Observational Studies in Epidemiology reporting guidelines.

### Measures

Table 1 presents the details of the measures used in this study.

**Table 1:** Descriptive statistics for depression, anxiety, stress, inadequate-self, hated-self, and reassured-self in 119 Czech students

Scales		*M*	SD	*α*
	Gender	93 females, 20 males, and 6 did not answer		
	Age (19–44 in our sample)	21.87	3.32	
Depression, Anxiety, and Stress Scale-21	Depression (0–42)	10.92	9.21	0.85
	Anxiety (0–42)	9.97	7.37	0.71
	Stress (0–42)	17.34	8.65	0.79
Forms of Self-Criticizing/Attacking & Self-Reassuring Scale	Inadequate-self (0–36)	17.31	8.17	0.89
	Hated-self (0–20)	3.88	4.20	0.83
	Reassured-self (0–32)	20.32	6.08	0.85

Depression, anxiety, and stress were measured using the Depression Anxiety and Stress Scale-21 (DASS-21), a shorter version of the original DASS-42 (Lovibond and Lovibond 1995), designed to measure negative emotions experienced in one’s daily life. The DASS-21 assesses the levels of depression, relating to hopelessness and devaluation of life (e.g., “I couldn’t seem to experience any positive feeling at all”), anxiety, relating to automatic arousal and situational anxiety (e.g., “I was worried about situations in which I might panic and make a fool of myself”), and stress, relating to chronic non-specific arousal (e.g., “I found it hard to wind down”). Participants are asked to mark how much each statement applied to them over the past week, on a scale of 0 to 3 (0 = “Did not apply to me at all”; 3 = “Applied to me very much, or most of the time”). The DASS21 subscales have good reliability (α ≧ 0.87) and validity (*r* = 0.46–0.85, compatible correlation coefficients with DASS-42—*r* = 0.42–0.84) (Antony et al. 1998).

Self-criticism and self-reassurance were assessed using the Forms of the Self-Criticizing/Attacking & Self-Reassuring Scale (FSCSR), evaluating how people relate to themselves when things go wrong for them (Gilbert et al. 2004). This 22-item scale consists of three parts: two forms of self-criticism (inadequate-self and hated-self) and one form of self-reassurance (reassured-self). Inadequate-self refers to a sense of personal inadequacy (e.g., “There is a part of me that puts me down”), hated-self to a desire to hurt or persecute the self (e.g., “I have a sense of disgust toward myself”), and reassured-self to a sense of self-compassion for the self (e.g., “I find it easy to forgive myself”). Participants respond to each item on a Likert scale of 0 to 4 (0 = “Not at all like me”; 4 = “Extremely like me”). The FSCSR subscales have good reliability (*α* = 0.90 for inadequate-self, *α* = 0.86 for hated-self, and *α* = 0.86 for reassured-self) and validity (|*r*| = 0.45–0.77) (Gilbert et al. 2004).

### Analytical Procedure

Cross-sectional design was employed. All collected data were first screened for outliers and the assumptions of parametric tests. Second, correlations between depression, anxiety, stress, self-criticism (inadequate-self and hated-self), and self-reassurance were evaluated, using IBM SPSS version 26. Finally, a series of path analyses were conducted to explore mediative effects of self-criticism and self-reassurance in the relationship between stress and depression/anxiety, using the Process Macro 3 for SPSS (Hayes 2013); 5000 bootstrapping re-samples and bias-corrected 95% CIs for indirect effects were applied.

## Results

Two scores in depression and one score in hated-self were identified as outliers using the outlier labeling rule (Hoaglin and Iglewicz 1987); therefore, those scores were winsorized (Tukey 1962). All variables had good reliability (*α* = 0.71–0.89).

### Relationships between Mental Health Problems, Self-Criticism, and Self-Reassurance

All variables except for reassured-self were not normally distributed (Shapiro–Wilk’s test, *p* < 0.05); therefore, they were square-root-transformed to satisfy the assumption of normality (Field 2017) (Table 2).

**Table 2:** Correlations between depression, anxiety, stress, inadequate-self, hated-self, and reassured-self in 119 Czech students

		1	2	3	4	5	6	7	8
1	Gender (0 = F, 1 = M)	–							
2	Age	0.25**	–						
3	Depression	− 0.07	− 0.01	–					
4	Anxiety	− 0.23*	0.07	0.50**	–				
5	Stress	− 0.10	0.001	0.63**	0.58**	–			
6	Inadequate-self	− 0.09	− 0.10	0.63**	0.51**	0.54**	–		
7	Hated-self	0.03	− 0.04	0.49**	0.49**	0.49**	0.70**	–	
8	Reassured-self	0.03	− 0.09	− 0.49**	− 0.38**	− 0.43**	− 0.62**	− 0.59**	–

*p < 0.05, **p < 0.01. For gender, point-biserial coefficients are reported

All the mental health problems (depression, anxiety, and stress) were positively associated with inadequate-self and hated-self while negatively associated with reassured-self.

### Mediative Effects on the Stress–Depression/Anxiety Relationships

Six combinations of path analyses were conducted using Model 4 in the Process macro (parallel mediation model; Hayes 2013) to examine whether inadequate-self, hated-self, and reassured-self (mediator variable) mediate the relationship between stress (predictor variable) and depression and anxiety (outcome variable), and their mediative effects.

#### Inadequate-Self Mediating Stress–Depression/Anxiety

**Fig. 1:**
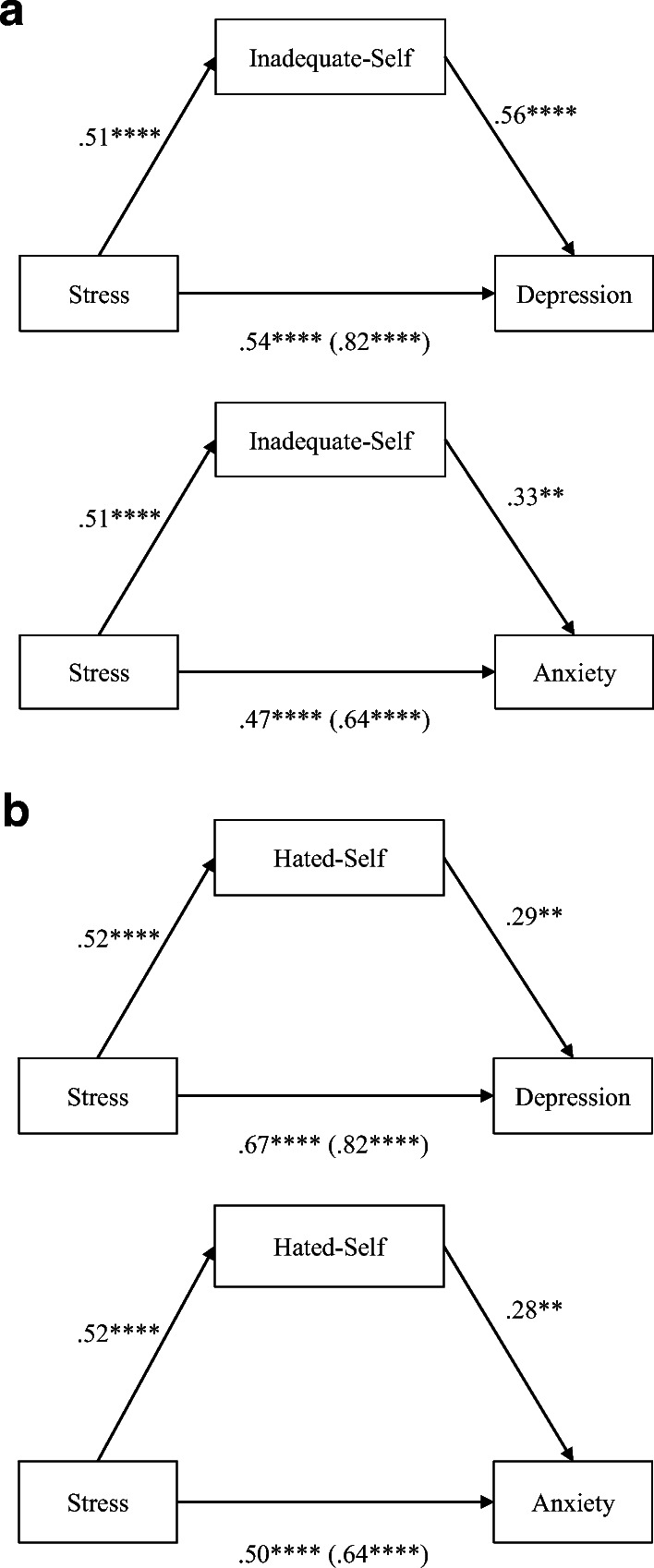
Parallel mediation model: stress as a predictor of depression/anxiety, mediated by self-criticism (inadequate-self and hated-self). **a** Inadequate-self. **b** Hated-self. Direct effect (total effect) from stress. Values attached to arrows are coefficients indicating impacts. *p < 0.05, **p < 0.01, ***p < 0.001, ****p < 0.0001

In both models, where depression or anxiety was the outcome variable, inadequate-self partially mediated the relationship between stress and depression/anxiety (Fig. 1a). The impact of inadequate-self on depression (*b* = 0.56, *t*(116) = 5.30, *p* < 0.001, CI [0.35, 0.77]) was greater than on anxiety (*b* = 0.33, *t*(116) = 3.29, *p* = 0.001, CI [0.13, 0.52]). Moreover, the indirect effects via inadequate-self explained 34% of the total effects between stress and depression, whereas the indirect effects explained 27% of the total effects between stress and anxiety.

#### Hated-Self Mediating Stress–Depression/Anxiety

In both depression and anxiety models, hated-self partially mediated the relationship between stress and depression/anxiety (Fig. 1b). The impacts of hated-self on depression (*b* = 0.29, *t*(116) = 2.96, *p* = 0.004, CI [0.10, 0.49]) and on anxiety (*b* = 0.28, *t*(116) = 3.22, *p* = 0.002, CI [0.11, 0.45]) were similar. Likewise, the rates of indirect to total effects were similar in both of the models: the indirect effects via hated-self explained 18% of the total effects between stress and depression, and 22% of the total effects between stress and anxiety.

#### Reassured-Self Mediating Stress–Depression/Anxiety

**Fig. 2:**
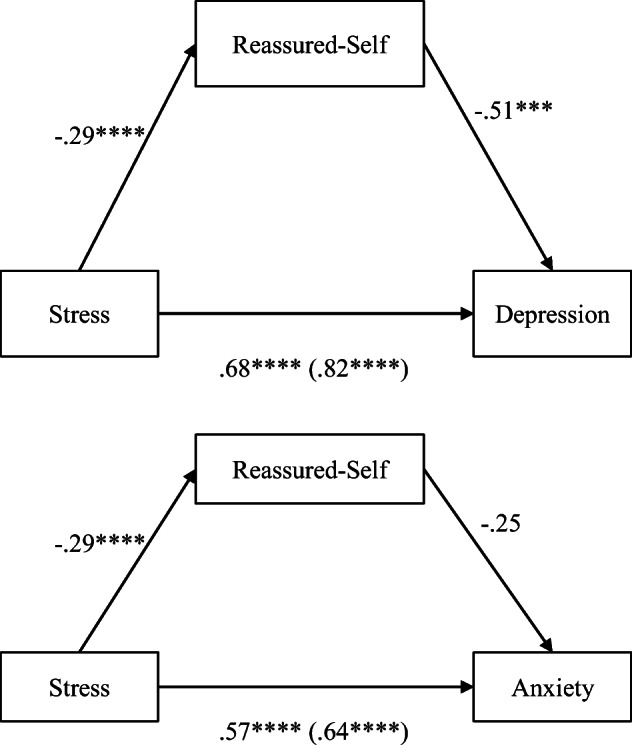
Parallel mediation model: stress as a predictor of depression, mediated by reassured-self. Direct effect (total effect) from stress. Values attached to arrows are coefficients indicating impacts. *p < 0.05, **p < 0.01, ***p < 0.001, ****p < 0.0001

Reassured-self partially mediated the stress-depression relationship (*b* = − 0.51, *t*(116) = − 3.43, *p* = 0.001, CI [− 0.80, − 0.21]), while it did not mediate the stress–anxiety relationship (*b* = − 0.25, *t*(116) = − 1.87, *p* = 0.06, CI [− 0.52, 0.01]; Fig. 2). The indirect effects via reassured-self from stress to depression was 17%.

Table 3 summarized the mediative effects of inadequate-self, hated-self, and reassured-self on the stress–depression and stress–anxiety relationships. Inadequate-self had greater impacts than hated-self and reassured-self.

**Table 3:** Summary of the mediative effects of inadequate-self, hated-self, and reassured-self on the stress–depression/anxiety relationship

**Mediator**	**Outcome**	**Effects on outcome (path b)**	**Indirect/total effects ratio (%)**
Inadequate-self	Depression	0.56****	34
	Anxiety	0.33**	27
Hated-self	Depression	0.29**	18
	Anxiety	0.28**	22
Reassured-self	Depression	− 0.58***	17
	Anxiety	− 0.25	–

**p < 0.01, ***p < 0.001, ****p < 0.0001

## Discussion

The current study was set out to investigate the mediating role of self-criticism and self-reassurance in the well-established pathway from stress to depression and anxiety. Our mediation model was based on the Transactional Model of Stress and Coping (Lazarus and Folkman 1984) evaluating the impacts of self-criticism and self-reassurance in the pathways between stress and psychopathology. Our results indicate that feelings of inadequacy significantly mediated the relationship between stress and depression, and accounted for over a third of this relationship, whereas similar but weaker results were found in the stress–anxiety relationship. By referring to this model, we established that individual attempts to cope with one’s feelings of inadequacy as well as the person–environment transaction can play an important role in developing stress and anxiety. Surprisingly, however, hated-self loaded to a lesser degree, which stands in contrast to previous findings that showed that hated-self was more strongly associated with depression than inadequate-self (Castilho et al. 2017; Gilbert et al. 2004; Werner et al. 2019). It is possible that the weaker association of hated-self is rooted in deliberate management of self-hate, relating to repressive coping, one feature of masculinity (Kotera et al. 2019), which is also a characteristic of the Czech culture (Hofstede 2001). Further research should investigate whether this finding is an artifact of our sample or a peculiarity within the general Czech culture. For example, a sample of university students from a culture that is distinct from the Czech culture can be explored to appraise the cultural impact of these relationships.

While anxiety is highly associated with depression in clinical and non-clinical samples (Sowislo and Orth 2013), it features empirically distinct symptoms and constructs from those in depression (McWilliams et al. 2001). This can be accounted by the fact that anxiety encompasses a wide range of conditions (disorders, phobias, stress reactions, etc.), and can therefore vary greatly in degree and severity. While the latter is also true of depression, depressive disorders and symptoms are generally associated with impaired psychological functioning, issues in relationships, work, physical wellbeing, and significantly elevated rates of suicidal behavior (Berman 2009; Räikkönen et al. 2007). This may explain why the impact of inadequate-self was greater on depression than anxiety, and contributed to the progression between stress and depression more strongly than between stress and anxiety. Following from this, the reassured-self was found to be partially significant in mediating the stress–depression relationship but not significant in mediating the stress–anxiety relationship. Our study indicates that the relation between the inadequate-self and reassured-self in depression is more symmetric than in anxiety, with significant or partial effects in both directions. As noted above, given the limitations of our sample, further research into the relationship between self-reassurance and anxiety would help understand whether the results hold in other cultural contexts, such as in Asian or African cultures.

Lastly, our findings can offer helpful implications to clinical practice. Treatment should focus on reducing inadequate-self of patients, rather than reducing hated-self or supporting reassured-self, in order to mitigate the levels of depression and anxiety when high stress is experienced. This is particularly pertinent during the current pandemic, where people’s stress levels are high in the Czech Republic (Trnka and Lorencova 2020). Interventions based on mindfulness, compassion, and attachment are deemed effective to reduce self-inadequacy (Barcaccia et al. 2020; Naismith et al. 2020). Educators and mental health practitioners in Czech universities may be able to protect students’ mental health by reducing self-inadequacy using these types of interventions. Intervention studies are recommended to evaluate the effects of these approaches for self-inadequacy and mental health problems in Czech university students.

### Limitations

While our findings offer helpful insights, several limitations should be noted. First, the participants were recruited through opportunity sampling at one university, which limits the generalizability of our findings. Second, self-report scales might limit their accuracy due to response biases (Kotera et al. 2020). Lastly, the causal direction of these effects has not been appraised. In the future, longitudinal data would help understand the temporal patterning of the relationships identified in this study and may help develop more effective interventions.

## Conclusion

The present study investigated the roles of self-reassurance and self-criticism in the progression between stress and psychopathology as illustrated in Transactional Model of Stress and Coping. As of yet, research investigating the association between self-criticism and psychopathology has primarily focused on investigating clinical samples (McIntyre et al. 2018; Werner et al. 2019). Furthermore, research investigating the role of self-reassurance in conjunction with self-criticism in depression and anxiety has been scarce (Sowislo and Orth 2013; Sherry et al. 2014). Our study extended existing literature by investigating the impact of self-reassurance and self-criticism in Czech undergraduate students, following the unnerving findings from the recent mental health study. Our findings suggest that inadequate-self was found to have a greater impact on depression than anxiety, contributing to the progression between stress and depression more strongly than between stress and anxiety. Our study indicates that the reassured-self is an important protective factor against depression but not anxiety. Finally, our study offers helpful implications to clinical practice by suggesting that treatment should focus on reducing patients’ inadequate-self in order to mitigate the levels of depression and anxiety. Future research in this area should focus on increasing the generalizability of findings by extending the sampling pool and investigating participants from other cultures.

## Data Availability

The data that support the findings of this study are available from the corresponding author, Y.K., on reasonable request.
